# Digital Competence Among Portuguese Nurses: Associations with Soft Skills

**DOI:** 10.3390/nursrep16090293

**Published:** 2026-08-23

**Authors:** Sérgio J. C. Figueiredo, Daniel J. Cunha, Graciele Oroski Paes, Mónica C. L. Araújo, Maria José S. Lumini Landeiro

**Affiliations:** 1 ULS de São João, 4200-319 Porto, Portugal; sergio.figueiredo@ulssjoao.min-saude.pt; 2 RISE-Health, Escola Superior de Enfermagem da Universidade do Porto, 4200-072 Porto, Portugal; danielcunha@esep.up.pt; 3 Escola de Enfermagem Anna Nery, Universidade Federal do Rio de Janeiro, Rio de Janeiro 20221-130, Brazil; gracieleoroski@eean.ufrj.br; 4 Enfermagem, Hospital da Luz Arrábida, 4400-123 Vila Nova de Gaia, Portugal; monica.lopes.araujo@hospitaldaluz.pt

**Keywords:** nursing, computer literacy, nursing informatics, professional competence, leadership, soft skills

## Abstract

**Background/Objectives**: Digital transformation is a strategic priority for healthcare systems, requiring nurses to develop competencies that enable the safe, effective, and critical use of digital technologies in clinical practice. Understanding nurses’ digital competence profile is essential to inform leadership, education, and workforce development strategies. This study aimed to assess digital competence among Portuguese nurses, examine its relationship with soft skills, and identify priority areas for professional development. **Methods**: A quantitative, descriptive-correlational, cross-sectional study was conducted with a nationally recruited convenience sample of Portuguese nurses. Data were collected using a Digital Competence Assessment Questionnaire based on the European Digital Competence Framework for Citizens (DigComp) and Soft Skills Inventory. Associations between digital competence and soft skills were analysed using descriptive, inferential, and multivariable statistical methods with IBM SPSS Statistics version 30.0. **Results**: Based on the exploratory classification of the knowledge/performance score, 52.0% of participants fell within the Intermediate and 27.2% within the Advanced proficiency intervals. Adapting and Coping, Analyzing and Interpreting, and Interacting and Presenting were positively associated with self-reflected digital competence. Multivariable analysis showed that soft skills accounted for a larger proportion of variance in self-reflected digital competence than in the knowledge/performance test score; however, the explanatory value of the latter model was limited. **Conclusions**: Participants were predominantly classified within the Intermediate and Advanced proficiency intervals, although comparatively lower descriptive performance was observed in Safety. The findings highlight the potential complementary role of technical competencies and soft skills in digital capability and suggest the value of further investigating targeted educational approaches. These findings may inform nursing leadership, education, and workforce-development strategies aimed at supporting digital competence and sustainable digital transformation.

## 1. Introduction

Digital transformation is reshaping healthcare systems through the increasing integration of electronic health records, telehealth, mobile health, artificial intelligence, clinical decision-support systems, and data-driven technologies. These developments are changing how healthcare professionals access information, communicate, make clinical decisions, and deliver care, while also introducing challenges related to information security, privacy, interoperability, and data governance [1,2,3,4,5,6]. As the largest professional group within the healthcare workforce, nurses play a central role in the implementation and safe use of digital technologies in clinical practice [1,3].

The World Health Organization has identified digital health as a strategic priority and advocates investment in a healthcare workforce equipped with appropriate digital competencies [2]. This perspective is consistent with the European Digital Competence Framework for Citizens (DigComp), which defines digital competence as the integrated set of knowledge, skills, and attitudes required for the critical, safe, and effective use of digital technologies. DigComp encompasses five competence areas: information and data literacy, communication and collaboration, digital content creation, safety, and problem solving [7,8,9]. Within healthcare, these competencies are increasingly relevant to safe, effective, and informed professional practice [2,4,5].

Nevertheless, assessing digital competence presents methodological challenges. Existing evidence shows considerable variability in how digital competence is conceptualized and measured among healthcare professionals [10,11,12]. In particular, measurement approaches rely extensively on self-assessment, which provides relevant information about professionals’ perceptions of their own abilities but does not necessarily capture the same aspects of competence as knowledge- or performance-based testing [12]. Self-reflection and knowledge/performance testing should therefore be regarded as complementary rather than interchangeable approaches to the assessment of digital competence.

Digital competence should also not be considered exclusively as a technical construct. Previous literature has highlighted the relevance of organisational and leadership contexts for digital transformation [13,14]. Broader competence frameworks also recognize transversal and behavioural competencies as relevant to professionals’ ability to adapt, collaborate, solve problems, and engage with digital technologies [9,12]. In this context, soft skills such as adaptability, critical thinking, communication, collaboration, and interpersonal interaction are recognised as relevant to professional performance in complex healthcare environments [15,16,17]. Digital soft skills have also been identified as potentially important for the healthcare workforce as digital technologies become increasingly embedded in professional practice [16].

Despite this conceptual relevance, the relationship between soft skills and digital competence among nurses remains insufficiently understood. Evidence concerning the digital competence development needs of healthcare professionals in the European context, including Portugal, also points to the need for context-sensitive professional development strategies [18]. However, little is known about how different soft-skills dimensions are associated with nurses’ digital competence in Portugal, particularly when self-reflected competence and knowledge/performance test results are considered separately.

Therefore, this study aimed to assess digital competence in a convenience sample of nurses recruited nationally in Portugal, examine its associations with soft skills, and identify priority areas for professional development. By considering both self-reflected digital competence and knowledge/performance test results, the study also sought to characterize complementary dimensions of digital competence and examine whether their associations with soft skills differed according to the assessment component.

## 2. Materials and Methods

### 2.1. Study Design

A quantitative, descriptive-correlational, cross-sectional study was conducted.

### 2.2. Population and Sample

The target population comprised registered nurses holding an active professional license and currently practicing in Portugal. The study sample included nurses from different healthcare settings and geographical regions who voluntarily completed an online questionnaire disseminated through the institutional communication channels of the Portuguese Order of Nurses, including its official website, institutional newsletter, and social media platforms. Because recruitment was conducted through an open online survey and information on the number of individuals who accessed or initiated the survey was not available, a response rate could not be calculated.

Data were collected between 6 June and 15 July 2025.

A non-probability convenience sampling strategy was adopted because equal selection probabilities across the target population could not be ensured. Participants were recruited based on availability and accessibility during the data collection period. All nurses who completed the questionnaire met the eligibility criteria, provided complete responses, and gave informed consent were included.

The final sample included 356 nurses.

Participants’ anonymity, confidentiality, and secure data processing were ensured in accordance with the General Data Protection Regulation (GDPR). Ethical approval was obtained from the Ethics Committee of the Nursing School of Porto (Reference No. CE_12/2025).

### 2.3. Instruments

Data were collected using a structured online questionnaire administered through Microsoft Forms. The questionnaire comprised three sections: (i) sociodemographic and professional characteristics, (ii) digital competence assessment, and (iii) soft skills assessment.

Sociodemographic and professional variables included gender, age group, years of professional experience, professional category, academic qualifications, healthcare setting, and geographical region of practice.

Digital competence was assessed using a questionnaire adapted from the European Commission’s Digital Skills Assessment Tool, which is based on the DigComp framework [19]. The official Portuguese-language version provided by the European Commission/Europass was used for all DigComp-based items; therefore, no translation of these items was performed by the authors. The questionnaire comprised two complementary components: a 10-item self-reflection component and a 23-item knowledge/performance test component.

The self-reflection component consisted of the 10 items included in the original Digital Skills Assessment Tool, with two items addressing each of the five DigComp competence areas. Each item used four hierarchically ordered response options reflecting increasing perceived autonomy in performing digital tasks, ranging from inability to perform the task to independent performance with the ability to support or guide others. The original response categories and scoring provided by the European Commission were retained. In the original adaptive assessment, these self-reflection items are used to generate an initial ability estimate that informs selection of the first knowledge/performance item. Once knowledge/performance items are administered within a competence area, the corresponding self-reflection responses are no longer included in the subsequent ability estimation.

The knowledge/performance test component used in the present study comprised a fixed set of 23 items selected from the original pool of 82 items included in the European Commission’s Digital Skills Assessment Tool. These items covered the five DigComp competence areas: (i) information and data literacy, (ii) communication and collaboration, (iii) digital content creation, (iv) safety, and (v) problem solving. Each item retained the competence-area mapping, proficiency level, response structure, and scoring information assigned in the original tool.

Selection of the 23 items was informed by a pilot administration of the original adaptive assessment involving five nurses purposively selected to provide diversity in academic background, professional experience, and clinical practice setting. Participants had between 5 and 20 years of professional experience and represented primary healthcare, private hospital care, and public hospital care, including community nursing, medical and surgical inpatient care, and operating room nursing. During these pilot administrations, the items presented to each participant were selected automatically by the adaptive algorithm implemented in the Europass platform. The authors did not define or modify the parameters governing this adaptive selection process.

According to the technical documentation provided by the European Commission, the adaptive Digital Skills Assessment Tool is based on Item Response Theory and uses a one-parameter logistic model for ability estimation and adaptive item selection. Item parameters are linked to the DigComp proficiency level assigned to each item and are used to estimate participants’ ability and determine the subsequent item to be presented. The adaptive procedure implemented by the Europass platform was used only during the pilot phase of the present study.

Following the five pilot administrations, the authors reviewed the items presented by the Europass adaptive system across participants. Items recurring across pilot administrations were prioritised, together with items required to ensure representation of all five DigComp competence areas. This process resulted in a fixed set of 23 knowledge/performance items. These same 23 items were subsequently administered to all participants in the main study, rather than allowing participant-specific item selection by the adaptive algorithm. This methodological adaptation was adopted to standardise item exposure and ensure that all participants completed the same set of questions. The distribution of the 23 selected items by assigned proficiency level and DigComp competence area is provided in Supplementary Table S1; because individual items could map to more than one competence area, area counts are not mutually exclusive.

For the author adapted 23 item fixed-form knowledge/performance test, each correctly answered item was assigned the proficiency value associated with its original difficulty/proficiency level: −1.38, −0.67, −0.21, +0.21, +0.67, and +1.38 for Levels 1–6, respectively. Incorrect responses were assigned the lower-bound score of −1.38; for Level 1 items, for which −1.38 represented the correct response value, an incorrect response was assigned −2.76 to preserve discrimination between correct and incorrect performance.

A mean-item level score was calculated for each participant. To incorporate the overall proportion of correct responses, an author developed proportional adjustment was applied as follows:

Proportional adjustment = [(1 − proportion of correct responses) × 2.76] − 1.38.

Final score = mean-item level score − proportional adjustment.

Because this fixed-form scoring procedure was not independently calibrated against the original computer adaptive assessment, application of the six DigComp derived proficiency thresholds was considered exploratory and used only as a descriptive framework. These classifications should not be interpreted as psychometrically equivalent to proficiency classifications generated by the original adaptive Digital Skills Assessment Tool.

For the knowledge/performance test component, the original item scoring, item-difficulty information, proficiency-level mappings, and ability-to-proficiency classification criteria provided by the European Commission were retained. Because the adaptive CAT procedure was replaced by a fixed-item administration in the main study, an additional proportional adjustment based on the overall proportion of correct responses was applied to the mean item-level score. This adjustment was developed by the authors specifically for the fixed-item administration used in the present study. The resulting score was subsequently classified into six proficiency levels derived from the DigComp framework [8]: Level 1 (Foundation), corresponding to a basic user performing simple tasks with guidance; Level 2 (Foundation), corresponding to a basic user performing simple tasks autonomously, with guidance when needed; Level 3 (Intermediate), corresponding to an independent user performing well-defined routine tasks and solving straightforward problems; Level 4 (Intermediate), corresponding to an independent user performing well-defined non-routine tasks according to individual needs; Level 5 (Advanced), corresponding to a user able to perform different tasks and guide others; and Level 6 (Advanced), corresponding to a user able to adapt to others in complex contexts.

Both the self-reflection and knowledge/performance test components were conceptually aligned with the five DigComp competence areas and interpreted using the same six-level DigComp proficiency framework. However, the two components differed in item format, number of items, and scoring procedure. Therefore, although the common DigComp framework provided a conceptual basis for comparing the resulting proficiency classifications within participants, the two classifications were not assumed to be psychometrically equivalent or independently cross-method calibrated.

In addition to the DigComp-based components, four multiple-choice knowledge items addressing Health Information Systems (HIS) were developed by the authors based on competency areas identified in the TIGER Initiative recommendations [20] and informed by subsequent work examining health information technology competencies in nursing informatics [21]. The items were adapted to the Portuguese nursing and healthcare context and addressed selected aspects of HIS knowledge. Each item had one predefined correct response. The items addressed distinct aspects of HIS knowledge and were not derived from a previously validated four-item scale.

For construction of the aggregate exploratory HIS knowledge index, responses were scored using predefined difficulty-weighted values assigned to the four items. Correct responses received the value associated with the assigned item difficulty, whereas incorrect responses were assigned the minimum scoring value of −1.38. A mean weighted item score was calculated and subsequently adjusted according to the overall proportion of correct responses using the proportional-adjustment procedure described above. The resulting score was used as an exploratory HIS knowledge index, without application of DigComp-derived proficiency classifications.

Soft skills were assessed using the Soft Skills Inventory, previously validated for the Portuguese population [17]. The instrument comprises 24 items distributed across eight dimensions:

- Adapting and Coping;
- Analyzing and Interpreting;
- Supporting and Cooperating;
- Creating and Conceptualizing;
- Enterprising and Performing;
- Leading and Deciding;
- Organizing and Executing;
- Interacting and Presenting.

Responses were recorded on a seven-point Likert scale ranging from 1 (“Never”) to 7 (“Always”). Mean scores were calculated for each dimension, with higher scores indicating a higher level of competency.

The original validation study reported satisfactory internal consistency, with Cronbach’s alpha coefficients ranging from 0.77 to 0.90 [17].

Although the original validation sample consisted of students, the instrument was selected for the present study because it assesses broad interpersonal and intrapersonal behavioural competencies that are conceptually relevant across professional contexts, including nursing practice.

### 2.4. Statistical Analysis

Statistical analyses were performed using IBM SPSS Statistics version 30.0 (IBM Corp., Armonk, NY, USA).

Descriptive statistics, Pearson’s product-moment correlations, and multiple linear regression models were used to examine digital competence and its associations with soft skills. To account for multiplicity in the 24 bivariate soft-skills correlation analyses, the Benjamini–Hochberg procedure was applied to control the false discovery rate (FDR).

Regression analyses were performed using continuous digital competence scores.

Assumptions underlying the multiple linear regression models were assessed through residual diagnostics and evaluation of linearity, homoscedasticity, and independence of residuals. Multicollinearity among predictors was assessed using variance inflation factors (VIF). For the four exploratory HIS knowledge items, correct-response rates, corrected item-total correlations, and KR-20 were examined.

Statistical significance was established at *p* < 0.05.

## 3. Results

### 3.1. Sample Characteristics

The study included 356 registered nurses. Most participants were female (87.1%, *n* = 310), aged between 41 and 50 years (41.6%, *n* = 148), and employed in public hospitals (60.1%, *n* = 214). Almost half of the sample comprised registered nurses (49.2%, *n* = 175), while 48.3% (*n* = 172) were specialist nurses and 2.5% (*n* = 9) were nurse managers.

Participants had a mean professional experience of 20.50 years (SD = 9.17; range 1–43 years). Most held a first-cycle degree (68.0%, *n* = 242), whereas 30.6% (*n* = 109) had completed a master’s degree and 1.4% (*n* = 5) held a doctoral degree. Additionally, 55.9% (*n* = 199) had completed postgraduate education. Detailed sociodemographic and professional characteristics are presented in Table 1.

### 3.2. Digital Competence

Self-reflected digital competence was assessed using the self-reflection component of the European Commission’s Digital Skills Assessment Tool. Most participants classified themselves at Intermediate proficiency levels (Levels 3–4; 44.6%, *n* = 159) or Advanced proficiency levels (Levels 5–6; 43.2%, *n* = 154), whereas only 12.1% classified themselves at Foundation proficiency levels (Levels 1–2).

The knowledge/performance test score ranged from −1.45 to 1.59, with a mean score of 0.09 (SD = 0.57), corresponding on average to Level 4 (Intermediate proficiency). Overall, 52.0% of participants were classified at Intermediate proficiency levels (Levels 3–4), 27.2% at Advanced proficiency levels (Levels 5–6), and 20.8% at Foundation proficiency levels (Levels 1–2).

The distribution of participants across the six DigComp proficiency levels according to the self-reflection and knowledge/performance test components is presented in Table 2.

### 3.3. Digital Competence Areas

Descriptive scores were calculated for the items mapped to each DigComp competence area. Mean scores were 0.28 (SD = 1.06) for Information and Data Literacy, 0.27 (SD = 0.66) for Communication and Collaboration, −0.01 (SD = 1.03) for Digital Content Creation, −0.12 (SD = 0.65) for Safety, and 0.08 (SD = 0.82) for Problem Solving.

These competence-area scores are presented for descriptive purposes only and should not be interpreted as evidence of relative strengths or weaknesses across competence areas.

Detailed results for each competence area are presented in Table 3.

Psychometric analysis of the four exploratory HIS knowledge items showed heterogeneous item performance and limited internal consistency (KR-20 = 0.36). Correct-response rates ranged from 16.3% to 90.7%, and corrected item-total correlations ranged from 0.10 to 0.31. The difficulty-weighted exploratory HIS knowledge index had a mean score of 0.16 (SD = 0.79). Given its exploratory nature and limited psychometric evidence, no DigComp-derived proficiency classification was assigned to this score.

### 3.4. Soft Skills

Descriptive analysis of the eight soft skills dimensions showed consistently high mean scores across all domains. The highest scores were observed for Enterprising and Performing (M = 5.67, SD = 0.83) and Interacting and Presenting (M = 5.65, SD = 0.79), whereas Analyzing and Interpreting presented the lowest mean score (M = 4.59, SD = 0.98). Descriptive statistics for all soft skills dimensions are presented in Table 4.

Internal consistency varied across the eight three-item soft-skills subscales, with Cronbach’s α ranging from 0.597 to 0.751 and McDonald’s ω from 0.607 to 0.755. Reliability coefficients for each subscale are reported in Supplementary Table S2. The complete intercorrelation matrix among the eight soft-skills dimensions is provided in Supplementary Table S3.

Although soft-skill scores were generally high, the proportion of participants achieving the maximum possible score ranged from 1.1% to 6.2% across subscales, providing no indication of a pronounced ceiling effect.

### 3.5. Association Between Digital Competence and Soft Skills

Correlation analyses revealed generally weak associations between soft skills and the knowledge/performance test score. Adapting and Coping was the only soft-skills dimension significantly associated with the knowledge/performance test score (*r* = 0.15, *p* = 0.004).

Exploratory correlations between the author-developed HIS knowledge index and the soft-skills dimensions were weak but statistically significant across the eight dimensions. The largest associations were observed for Adapting and Coping (*r* = 0.22, *p* < 0.001) and Analyzing and Interpreting (*r* = 0.22, *p* < 0.001).

Self-reflected digital competence showed consistently stronger associations with soft skills, ranging from weak to moderate. The strongest associations were observed for Analyzing and Interpreting (*r* = 0.38, *p* < 0.001), Adapting and Coping (*r* = 0.37, *p* < 0.001), and Interacting and Presenting (*r* = 0.32, *p* < 0.001). Complete correlation coefficients are presented in Table 5.

The overall pattern of statistical significance remained unchanged after Benjamini–Hochberg FDR adjustment.

### 3.6. Multivariable Associations with Digital Competence

Two multiple linear regression models were used to examine multivariable associations between soft skills and digital competence. For the knowledge/performance test score, the overall model was statistically significant (F(8,347) = 2.926, *p* = 0.004), but its explanatory value was limited (R^2^ = 0.063; adjusted R^2^ = 0.042). After adjustment for the other soft-skills dimensions included in the model, Adapting and Coping showed a significant positive association (β = 0.276, *p* = 0.009), whereas Creating and Conceptualizing showed a significant negative association (β = −0.306, *p* = 0.001).

For self-reflected digital competence, the model accounted for 22.1% of the variance (R^2^ = 0.221; adjusted R^2^ = 0.203) and was statistically significant (F(8,347) = 12.335, *p* < 0.001). After adjustment for the other soft-skills dimensions included in the model, Analyzing and Interpreting (β = 0.342, *p* < 0.001), Adapting and Coping (β = 0.259, *p* = 0.008), and Interacting and Presenting (β = 0.246, *p* = 0.004) showed significant positive associations, whereas Creating and Conceptualizing showed a significant negative association (β = −0.218, *p* = 0.011). Regression model parameters, including unstandardized coefficients, standard errors, 95% confidence intervals, tolerance, and variance inflation factors, are presented in Table 6.

## 4. Discussion

This study examined digital competence and its associations with soft skills in a convenience sample of nurses recruited nationally in Portugal. Digital competence was assessed using complementary self-reflection and knowledge/performance test components. Overall, participants in this study demonstrated an intermediate to advanced level of digital competence, with more than three-quarters of participants classified at Intermediate or Advanced proficiency levels. These findings are consistent with recent evidence indicating that healthcare professionals possess digital competencies that extend beyond the basic use of technologies, enabling them to integrate digital tools into clinical practice and adapt their use to patient care needs [10,22].

Descriptive variation was observed across the scores obtained from items mapped to the different DigComp competence areas. When the six-level numerical thresholds were applied exploratorily, Information and Data Literacy, Communication and Collaboration, and Problem Solving fell within the Level 4 interval, whereas Digital Content Creation and Safety fell within the Level 3 interval. However, these classifications should not be interpreted as calibrated differences in domain-specific proficiency.

The descriptive patterns observed for Digital Content Creation and Safety should therefore be regarded as hypothesis-generating findings that warrant further assessment rather than as confirmed competency deficits. Safety is of particular relevance for future investigation because cybersecurity, data protection, privacy, and secure digital practice are recognised as important requirements of contemporary digital healthcare [23,24]. However, independently calibrated domain-specific assessments are required to determine whether the descriptive patterns observed in the present study correspond to genuine differences in competence.

Although both components were conceptually anchored to the same five DigComp competence areas and six-level proficiency framework, they differed in item format, number of items, and scoring procedure and were not independently cross-method calibrated. Accordingly, their proficiency classifications were interpreted separately and should not be regarded as directly comparable or interchangeable measures. Self-reflection and knowledge/performance testing may instead provide complementary information about digital competence, as they capture related but non-equivalent aspects of the construct.

Moreover, the two methods were not independently cross-method calibrated. Consequently, the descriptive differences between the self-reflection and knowledge/performance classifications should be interpreted cautiously and should not be regarded as direct evidence that participants systematically overestimated their digital competence. Part of the observed discrepancy may reflect the non-equivalence of the two assessment components, which capture related but distinct aspects of digital competence. Similar discrepancies have been reported in recent studies of digital literacy among healthcare professionals, particularly when competence is assessed using self-report instruments [25]. This observation highlights the value of considering complementary assessment approaches when evaluating digital competence, while recognizing that self-reflection and knowledge/performance testing are not interchangeable measures of the construct.

Taken together, these results highlight the importance of adopting comprehensive assessment strategies that integrate self-reflection and knowledge/performance test components, thereby capturing complementary aspects of digital competence [25]. This approach is supported by evidence showing that digital competence is a multidimensional construct encompassing technical abilities, competencies related to Health Information Systems, and ethical and legal aspects of digital practice. Consequently, its assessment requires psychometrically robust and multidimensional instruments capable of capturing these complementary dimensions [26].

The approximately one-fifth of participants whose knowledge/performance scores fell within the numerical interval corresponding to Foundation proficiency warrants further investigation. Given the exploratory nature of these classifications, independently validated assessments are needed to determine whether specific educational needs exist and which competence areas should be prioritised. Previous evidence similarly highlights the importance of assessing digital competence development needs and designing context-sensitive educational strategies in healthcare [18,27].

### 4.1. The Role of Soft Skills in Nurses’ Digital Competence

One of the most distinctive contributions of this study was the simultaneous examination of digital competence and soft skills, providing a broader understanding of the factors associated with nurses’ engagement with digital technologies. Overall, participants demonstrated high levels of soft skills across most dimensions, particularly Enterprising and Performing, Interacting and Presenting, and Adapting and Coping. These competencies reflect behavioural characteristics such as proactivity, interpersonal effectiveness, and behavioural flexibility, which are increasingly recognised as essential for professional practice in complex and rapidly evolving healthcare environments. This pattern is consistent with recent evidence identifying adaptability, communication, creativity, and results orientation as key competencies for nursing practice in the digital era [15,28].

The relationship between soft skills and digital competence varied according to the assessment method used.

Importantly, the multivariable model for the knowledge/performance test score had limited explanatory value, accounting for only 6.3% of the observed variance (adjusted R^2^ = 4.2%). Accordingly, the adjusted associations observed for individual soft-skills dimensions should be interpreted cautiously and should not be regarded as evidence that these competencies are major determinants of tested digital competence. The limited explained variance indicates that a substantial proportion of variability in knowledge/performance test scores is associated with factors not captured by the soft-skills dimensions included in the model.

For the knowledge/performance test score, Adapting and Coping was the only soft-skill dimension showing a significant positive bivariate association with digital proficiency. In the multivariable model, Adapting and Coping showed a positive adjusted association, whereas Creating and Conceptualizing showed a negative adjusted association.

The negative adjusted coefficients observed for Creating and Conceptualizing should be interpreted cautiously. Their divergence from the corresponding bivariate associations, together with the substantial intercorrelations among the soft-skills dimensions, is consistent with a suppression or contextual effect arising from shared variance among predictors. Accordingly, these negative coefficients should not be interpreted as evidence that higher Creating and Conceptualizing competence is independently associated with lower digital competence. These findings should therefore be interpreted as associations rather than evidence of causal or predictive effects. This association suggests that behavioural flexibility may be relevant to how professionals engage with digital technologies and adapt to technological innovation and evolving work processes. This interpretation is supported by previous studies highlighting adaptability as a key competence for responding effectively to technological and organisational change in healthcare settings [29,30].

The exploratory HIS knowledge index showed weak positive associations with the soft-skills dimensions. These findings may suggest potential relationships between selected HIS knowledge indicators and transversal competencies; however, they should be interpreted cautiously given the limited internal consistency and exploratory nature of the four-item index. Rather than providing evidence of established relationships with a validated HIS competence construct, these findings should be considered hypothesis-generating and require replication using a validated and more comprehensive measure of HIS competence.

In contrast, self-reflected digital competence showed consistently stronger associations with soft skills than the knowledge/performance test score. Moderate correlations were identified for Analyzing and Interpreting, Adapting and Coping, and Interacting and Presenting, indicating that professionals’ perceptions of their digital competence are closely related to behavioural and interpersonal characteristics. This finding suggests that self-reflected digital competence may be associated not only with technical proficiency but also with broader personal attributes, including confidence, critical thinking, communication, and adaptability.

These associations should, however, be interpreted with caution. Because both soft skills and self-reflected digital competence were assessed through self-report, part of their covariance may reflect shared-method variance, including common response tendencies and social-desirability effects, rather than exclusively substantive relationships between the constructs. In addition, although soft-skill scores were generally high, the low proportion of participants achieving the maximum possible scores did not indicate a pronounced ceiling effect. Nevertheless, the concentration of scores toward the upper range of several dimensions may have restricted variability and should be considered when interpreting the magnitude of the observed associations.

This interpretation is consistent with previous evidence indicating associations between digital competencies and a combination of technical, behavioural, and contextual factors. A systematic review identified individual and team-related characteristics as factors associated with nurses’ adoption of digital health technologies [31], while a subsequent scoping review highlighted the multifactorial nature of digital health competencies among healthcare professionals [32]. More recently, a cross-sectional study identified significant associations between individual and contextual factors and digital health competence [30]. Collectively, these findings support the interpretation that behavioural characteristics are associated with how healthcare professionals perceive and engage with digital technologies.

Overall, the present findings indicate stronger associations between soft skills and self-reflected digital competence than between soft skills and the knowledge/performance test score. The limited explanatory value of the knowledge/performance model suggests that variation in tested digital competence is largely associated with factors not captured by the soft-skills dimensions examined here. This pattern highlights the importance of considering both technical and non-technical competencies when designing educational programmes and strategies aimed at strengthening nurses’ digital capability, while recognizing that the cross-sectional design does not permit causal or directional interpretations.

### 4.2. Implications for Nursing Management, Leadership, and Education

The findings of this study have important implications for human resource management in healthcare, particularly in the context of the ongoing digital transformation of healthcare systems. Although participants in this study generally demonstrated intermediate levels of digital competence, the descriptive domain-level patterns observed in this study may help identify areas warranting further investigation in subsequent training-needs assessments. Given the recognised importance of cybersecurity, privacy, data protection, and safe digital practice in healthcare [23,24], Safety represents a particularly relevant domain for further assessment, although the present descriptive findings alone do not establish a specific training deficit. The exploratory HIS findings also highlight the value of further investigating nurses’ knowledge of health information systems using more comprehensive and independently validated assessment instruments.

These results suggest that the potential value of integrating digital competence development with transversal and interpersonal competencies warrants further investigation. As healthcare becomes increasingly digitalized, professionals are expected not only to use digital technologies effectively, but also to apply them critically, safely, and appropriately within complex and constantly evolving clinical environments. This perspective is consistent with current evidence emphasizing that successful digital transformation depends not only on technological implementation but also on the continuous development of healthcare professionals’ competencies [3,18].

The observed associations suggest that the potential role of behavioural competencies, including Adapting and Coping, Analyzing and Interpreting, and Interacting and Presenting, in digital competence development warrants further investigation. Longitudinal and intervention studies are needed to determine whether educational strategies combining digital competence and soft-skills development result in improvements in knowledge/performance-assessed digital competence. This integrated approach is supported by previous studies highlighting the importance of organisational environments that promote continuous learning, motivation, and competence development as essential conditions for successful digital transformation [11,18,22].

From a leadership perspective, the competency patterns observed in the present study have potential implications for nurse managers and healthcare organizations. Although leadership was not directly assessed, existing evidence identifies nurse managers as important facilitators of digital transformation, including through the promotion of continuous professional development, organisational learning, change management, and the safe and ethical use of digital technologies [13,14,33,34]. Accordingly, the present findings may help inform areas for further workforce assessment, while leadership and organisational strategies can support broader digital competence development through continuing education, organisational learning, and the safe and ethical use of digital technologies. The present results are also consistent with international evidence emphasizing that successful digital transformation in healthcare is associated not only with technological infrastructure but also with organisational culture, leadership, workforce preparedness, and competency development [1,14,35]. Therefore, digital transformation strategies should incorporate workforce development as a central component, ensuring equitable access to digital education, reducing disparities in digital competence, and fostering organisational cultures that support innovation, collaboration, and continuous quality improvement [12,36].

Taken together with the existing literature, the present findings support consideration of digital competence within human resource management and workforce-development policies. Aligning professional development policies, leadership strategies, and organisational priorities with national and international digital health agendas will be essential to strengthen workforce capability, improve patient safety, and promote sustainable digital transformation across healthcare systems [37].

### 4.3. Study Limitations and Future Research

This study has several limitations that should be considered when interpreting the findings. First, the use of a non-probability convenience sample based on voluntary participation may have introduced selection bias, potentially favouring nurses with greater interest in digital health or technology-related topics. The non-probability sampling strategy does not support statistical representativeness of all nurses practicing in Portugal, and the findings should therefore not be generalised to the entire Portuguese nursing population.

Second, the adaptive algorithm originally incorporated into the European Commission’s Digital Skills Assessment Tool could not be implemented in the main study, requiring the use of an author-adapted 23-item fixed-form knowledge/performance test. Item selection was informed by adaptive pilot administrations involving only five nurses, all of whom demonstrated intermediate-to-advanced digital proficiency, with no participants representing basic proficiency levels. This may have limited the representation of lower digital literacy profiles in the fixed-form item set. Moreover, the fixed-form test was not independently calibrated against the original adaptive Digital Skills Assessment Tool. Although the selected items retained their original difficulty/proficiency assignments, the fixed-form administration and author-developed proportional adjustment do not establish equivalence with the original adaptive IRT-based scoring procedure. Internal consistency of the 23-item fixed form was limited, and item-level discrimination was heterogeneous. Sensitivity analyses indicated that the main association findings were robust to alternative scoring specifications; however, proficiency-level classifications were sensitive to the scoring method. Accordingly, the six DigComp-derived proficiency classifications should be interpreted as exploratory rather than as equivalent to classifications generated by the original adaptive assessment. This limitation also applies to the domain-level analyses. Further psychometric validation of the fixed-form test in larger and more heterogeneous nursing samples is therefore warranted. The four author-developed HIS items showed limited internal consistency and were not independently validated as a unidimensional measure of HIS competence; findings based on the exploratory HIS knowledge index should therefore be interpreted with caution.

Third, although the Soft Skills Inventory assesses broad interpersonal and intrapersonal competencies relevant to nursing practice, its original validation was conducted in a student population. The substantial intercorrelations observed among the eight subscales indicate considerable overlap between dimensions in the present sample, and further psychometric validation in nursing and healthcare professional populations is warranted. Moreover, the modest internal consistency observed for some three-item subscales may introduce measurement error, potentially attenuating bivariate associations and contributing to uncertainty in individual regression coefficients. In addition, the self-reported nature of the soft-skills assessment may be susceptible to social-desirability bias and shared-method variance, particularly in analyses involving self-reflected digital competence. The multivariable soft-skills models were not adjusted for potential sociodemographic and professional confounders; therefore, residual confounding cannot be excluded.

Finally, organisational factors such as digital infrastructure, institutional digital culture, leadership styles, and organisational support for digital transformation were not examined, despite their potential relevance to nurses’ digital competence.

Future research should adopt longitudinal designs to better understand the development of digital competence over time, further validate the measurement instruments in nursing and healthcare professional populations, incorporate organisational factors associated with digital capability, and evaluate the effectiveness of educational and organisational interventions aimed at strengthening both digital competence and soft skills across different healthcare settings.

## 5. Conclusions

The findings provide a descriptive profile of digital competence among the study participants, while proficiency classifications derived from the author-adapted fixed-form assessment should be interpreted as exploratory. Areas showing comparatively lower descriptive scores, including Digital Content Creation and Safety, warrant further investigation using independently calibrated domain-specific measures.

The findings also indicate associations between digital competence and transversal competencies, particularly soft skills, although these relationships varied according to the assessment component. Soft skills showed stronger associations with self-reflected digital competence than with the knowledge/performance test score. However, the limited explanatory value of the knowledge/performance model indicates that most of the variation in tested digital competence was not accounted for by the soft-skills dimensions examined in this study.

These findings support differentiated, continuous, and evidence-based training strategies that integrate digital competence and soft-skills development while responding to the demands of clinical practice. They also have potential implications for nursing leadership and human resource management, particularly in guiding training and professional-development strategies. However, leadership was not evaluated in this study, and its relationship with digital competence should therefore be examined directly in future research.

From a strategic perspective, digital capability should be considered within human resource management policies in healthcare, with training and professional-development strategies aligned with the evolving demands of digital health.

## Figures and Tables

**Table 1:** Characteristics of participants.

Characteristic	Frequency (*n*)		%	
Gender				
Female	310		87.1%	
Male	46		12.9%	
Age group (years)				
20–30	32		9.0%	
31–40	91		25.6%	
41–50	148		41.6%	
51–60	77		21.6%	
61–70	8		2.2%	
Professional category				
Registered Nurse	175		49.2%	
Specialist Nurse	172		48.3%	
Nurse Manager	9		2.5%	
Practice setting				
Public hospital	214		60.1%	
Primary Health Care	78		21.9%	
Private hospital	24		6.7%	
Residential care facility	4		1.1%	
National Network of Integrated Continuing Care	8		2.2%	
Other	28		7.9%	
Geographical region				
North	189		53.1%	
Centre	90		25.3%	
South	55		15.4%	
Azores	11		3.1%	
Madeira	11		3.1%	
Academic qualification				
First-cycle degree	242		68.0%	
Master’s degree	109		30.6%	
Doctoral degree	5		1.4%	
Postgraduate education				
Yes	199		55.9%	
No	157		44.1%	
Total	356		100%	
Professional experience (years)	Minimum	Maximum	Mean (M)	SD
	1	43	20.50	9.17

**Table 2:** Digital Competence.

Exploratory Descriptive Level	Self-Reflection Component *n* (%)	Knowledge/Performance Test Component *n* (%)
Level 1 (Foundation)	11 (3.1)	4 (1.1)
Level 2 (Foundation)	32 (9.0)	70 (19.7)
Level 3 (Intermediate)	56 (15.7)	90 (25.3)
Level 4 (Intermediate)	103 (28.9)	95 (26.7)
Level 5 (Advanced)	67 (18.8)	72 (20.2)
Level 6 (Advanced)	87 (24.4)	25 (7.0)
Total	356 (100.0)	356 (100.0)
Mediana (IQR)	4 (3–5)	4 (3–5)

Note. Proficiency levels were classified according to the scoring criteria of the European Commission’s Digital Skills Assessment Tool: Level 1 (≤−0.97), Level 2 (>−0.97 to ≤−0.43), Level 3 (>−0.43 to ≤0.00), Level 4 (>0.00 to ≤0.43), Level 5 (>0.43 to ≤0.97), and Level 6 (>0.97).

**Table 3:** Digital Competence Areas.

Digital Competence Area	Mean	SD
Information and Data Literacy	0.28	1.06
Communication and Collaboration	0.27	0.66
Digital Content Creation	−0.01	1.03
Safety	−0.12	0.65
Problem Solving	0.08	0.82

**Table 4:** Soft Skills.

Soft Skills Dimension	Mean	SD
Adapting and Coping	5.37	0.87
Analyzing and Interpreting	4.59	0.98
Supporting and Cooperating	5.46	0.91
Creating and Conceptualizing	5.45	0.87
Enterprising and Performing	5.67	0.83
Leading and Deciding	5.39	0.92
Organizing and Executing	5.39	0.96
Interacting and Presenting	5.65	0.79

**Table 5:** Associations Between Digital Competence and Soft Skills.

Soft Skills Dimension	Knowledge/Performance Test Score			Exploratory HIS Knowledge Index			Self-Reflected Digital Competence		
	*r*	*p*	BH-FDR Adjusted *p*	*r*	*p*	BH-FDR Adjusted *p*	*r*	*p*	BH-FDR Adjusted *p*
Adapting and Coping	0.15	0.004	0.007	0.22	<0.001	<0.001	0.37	<0.001	<0.001
Analyzing and Interpreting	0.09	0.098	0.131	0.22	<0.001	<0.001	0.38	<0.001	<0.001
Supporting and Cooperating	0.08	0.136	0.153	0.13	0.015	0.022	0.27	<0.001	<0.001
Creating and Conceptualizing	−0.03	0.563	0.561	0.14	0.008	0.013	0.18	<0.001	0.002
Enterprising and Performing	0.05	0.306	0.324	0.13	0.015	0.022	0.19	<0.001	0.001
Leading and Deciding	0.08	0.131	0.153	0.17	0.002	0.004	0.26	<0.001	<0.001
Organizing and Executing	0.06	0.300	0.324	0.15	0.005	0.009	0.23	<0.001	<0.001
Interacting and Presenting	0.09	0.083	0.109	0.18	<0.001	0.002	0.32	<0.001	<0.001

Note. *r* = Pearson’s product-moment correlation coefficient. *p* values were adjusted for multiple testing across the 24 bivariate correlations using the Benjamini–Hochberg false discovery rate procedure. Associations involving the exploratory HIS knowledge index should be interpreted cautiously given its limited psychometric evidence.

**Table 6:** Multivariable Associations with Digital Competence.

Soft Skills Dimension	Knowledge/Performance Test Score						
	B	SE	β	95% CI for B	*p*	Tolerance	VIF
Adapting and Coping	0.183	0.070	0.276	[0.045, 0.320]	0.009	0.242	4.13
Analyzing and Interpreting	−0.003	0.054	−0.006	[−0.109, 0.102]	0.949	0.323	3.09
Supporting and Cooperating	0.019	0.059	0.030	[−0.097, 0.135]	0.749	0.305	3.28
Creating and Conceptualizing	−0.202	0.062	−0.306	[−0.324, −0.081]	0.001	0.311	3.21
Enterprising and Performing	−0.032	0.068	−0.047	[−0.166, 0.101]	0.634	0.281	3.56
Leading and Deciding	0.061	0.071	0.098	[−0.079, 0.202]	0.393	0.207	4.83
Organizing and Executing	−0.015	0.059	−0.025	[−0.132, 0.102]	0.802	0.270	3.70
Interacting and Presenting	0.046	0.068	0.064	[−0.088, 0.180]	0.498	0.308	3.24
**Soft Skills Dimension**	**Self-Reflected Digital Competence**						
	**B**	**SE**	**β**	**95% CI for B**	***p***	**Tolerance**	**VIF**
Adapting and Coping	0.069	0.026	0.259	[0.019, 0.120]	0.008	0.242	4.13
Analyzing and Interpreting	0.081	0.020	0.342	[0.042, 0.120]	<0.001	0.323	3.09
Supporting and Cooperating	0.018	0.022	0.073	[−0.024, 0.061]	0.398	0.305	3.28
Creating and Conceptualizing	−0.058	0.023	−0.218	[−0.103, −0.014]	0.011	0.311	3.21
Enterprising and Performing	−0.049	0.025	−0.176	[−0.099, 0.000]	0.0503	0.281	3.56
Leading and Deciding	−0.011	0.026	−0.042	[−0.063, 0.041]	0.686	0.207	4.83
Organizing and Executing	−0.023	0.022	−0.095	[−0.066, 0.020]	0.299	0.270	3.70
Interacting and Presenting	0.072	0.025	0.246	[0.023, 0.122]	0.004	0.308	3.24

Note. B = unstandardized regression coefficient; SE = standard error; β = standardized regression coefficient; CI = confidence interval; VIF = variance inflation factor. Tolerance and VIF values are identical across models because both models include the same eight predictors. Statistically significant coefficients were defined using *p* < 0.05. Knowledge/Performance Test Score: R^2^ = 0.063; adjusted R^2^ = 0.042; F(8,347) = 2.926; *p* = 0.004. Self-Reflected Digital Competence: R^2^ = 0.221; adjusted R^2^ = 0.203; F(8,347) = 12.335; *p* < 0.001.

## Data Availability

The datasets generated and analysed during the current study are not publicly available due to ethical and privacy restrictions related to participant confidentiality.

## Supplementary Materials

The following supporting information can be downloaded at: https://www.mdpi.com/article/10.3390/nursrep16090293/s1, Table S1. Distribution of Items in the Author-Adapted 23-Item Fixed-Form Knowledge/Performance Test; Table S2. Internal Consistency of the Soft Skills Inventory Subscales in the Present Sample; Table S3. Spearman Intercorrelations and Descriptive Statistics for the Soft-Skills Dimensions.

## Public Involvement Statement

No public involvement in any aspect of this research.

## Guidelines and Standards Statement

This manuscript was prepared in accordance with the Strengthening the Reporting of Observational Studies in Epidemiology (STROBE) Statement for cross-sectional studies.
