# Supplementary material for: Digital Competence Among Portuguese Nurses: Associations with Soft Skills
Source: Nurs Rep. 2026 Aug 23;16(9):293. doi: 10.3390/nursrep16090293 (PMC13610122; doi:10.3390/nursrep16090293)
Supplement: Supplementary file 1 [file nursrep-16-00293-s001.zip › nursrep-4483580-supplementary.pdf]

Supplementary Table S1. Distribution of Items in the Author-Adapted 23-Item Fixed-Form Knowledge/Performance Test

Panel A. Distribution by Assigned Proficiency Level

Assigned proficiency level	n (%)
Level 1	1 (4.3)
Level 2	1 (4.3)
Level 3	2 (8.7)
Level 4	7 (30.4)
Level 5	9 (39.1)
Level 6	3 (13.0)
Total	23 (100.0)

Panel B. Coverage by DigComp Competence Area

DigComp competence area	Number of items mapped to the area, n
Information and Data Literacy	6
Communication and Collaboration	9
Digital Content Creation	5
Safety	12
Problem Solving	12

Note. Panel A presents the distribution of the 23 items according to their assigned proficiency level. In Panel B, individual items could map to more than one DigComp competence area; therefore, competence-area counts are not mutually exclusive and do not sum to 23. Item-level content and detailed item-to-area mappings are not reproduced to preserve the confidentiality of the assessment materials provided by the European Commission.

Supplementary Table S2. Internal Consistency of the Soft Skills Inventory Subscales in the Present Sample

Soft-skills dimension	Items	Cronbach's α	McDonald's ω
Adapting and Coping	3	0.695	0.706
Analyzing and Interpreting	3	0.597	0.607
Supporting and Cooperating	3	0.707	0.716
Creating and Conceptualizing	3	0.726	0.752
Enterprising and Performing	3	0.674	0.697
Leading and Deciding	3	0.751	0.755
Organizing and Executing	3	0.666	0.688
Interacting and Presenting	3	0.694	0.701

Note. α = Cronbach's alpha; ω = McDonald's omega. Each subscale comprised three items. N = 356.

Supplementary Table S3. Spearman Intercorrelations and Descriptive Statistics for the Soft-Skills Dimensions

Soft Skills Dimension	1	2	3	4	5	6	7	8	Mean	SD	% maximum (7)
1-Adapting and Coping	—								5.37	0.87	3.9%
2-Analyzing and Interpreting	0.760*	—							4.59	0.98	1.1%
3-Supporting and Cooperating	0.671*	0.595*	—						5.46	0.91	5.1%
4-Creating and Conceptualizing	0.664*	0.648*	0.674*	—					5.45	0.87	3.7%
5-Enterprising and Performing	0.725*	0.601*	0.670*	0.718*	—				5.67	0.83	6.2%
6-Leading and Deciding	0.741*	0.697*	0.785*	0.771*	0.744*	—			5.39	0.92	5.9%
7-Organizing and Executing	0.726*	0.729*	0.635*	0.719*	0.766*	0.765*	—		5.39	0.96	5.6%
8-Interacting and Presenting	0.715*	0.569*	0.728*	0.704*	0.683*	0.734*	0.669*	—	5.65	0.79	5.3%

Note. Values below the diagonal are Spearman's rank-order correlation coefficients (ϱ). "% maximum (7)" indicates the percentage of participants obtaining the maximum possible score of 7. N = 356.
* $p<0.0001$.
